# Automatic quantification of retinal photoreceptor integrity to predict persistent disease activity in neovascular age-related macular degeneration using deep learning

**DOI:** 10.3389/fnins.2022.952735

**Published:** 2022-08-18

**Authors:** Xian Song, Qian Xu, Haiming Li, Qian Fan, Yefeng Zheng, Qiang Zhang, Chunyan Chu, Zhicheng Zhang, Chenglang Yuan, Munan Ning, Cheng Bian, Kai Ma, Yi Qu

**Affiliations:** ^1^Department of Geriatrics, Qilu Hospital of Shandong University, Jinan, China; ^2^Tencent Healthcare, Shenzhen, China; ^3^Xiaohe Healthcare, ByteDance, Guangzhou, China

**Keywords:** neovascular age-related macular degeneration, image analysis, deep learning, optical coherence tomography, anti-VEGF therapy, retinal photoreceptor

## Abstract

**Purpose:**

Using deep learning (DL)-based technique, we identify risk factors and create a prediction model for refractory neovascular age-related macular degeneration (nAMD) characterized by persistent disease activity (PDA) in spectral domain optical coherence tomography (SD-OCT) images.

**Materials and methods:**

A total of 671 typical B-scans were collected from 186 eyes of 186 patients with nAMD. Spectral domain optical coherence tomography images were analyzed using a classification convolutional neural network (CNN) and a fully convolutional network (FCN) algorithm to extract six features involved in nAMD, including ellipsoid zone (EZ), external limiting membrane (ELM), intraretinal fluid (IRF), subretinal fluid (SRF), pigment epithelium detachment (PED), and subretinal hyperreflective material (SHRM). Random forest models were probed to predict 1-year disease activity (stable, PDA, and cured) based on the quantitative features computed from automated segmentation and evaluated with cross-validation.

**Results:**

The algorithm to segment six SD-OCT features achieved the mean accuracy of 0.930 (95% CI: 0.916–0.943), dice coefficients of 0.873 (95% CI: 0.847–0.899), a sensitivity of 0.873 (95% CI: 0.844–0.910), and a specificity of 0.922 (95% CI: 0.905–0.940). The six-metric model including EZ and ELM achieved the optimal performance to predict 1-year disease activity, with an area under the receiver operating characteristic (ROC) curve (AUC) of 0.980, the accuracy of 0.930, the sensitivity of 0.920, and the specificity of 0.962. The integrity of EZ and ELM significantly improved the performance of the six-metric model than that of the four-metric model.

**Conclusion:**

The prediction model reveals the potential to predict PDA in nAMD eyes. The integrity of EZ and ELM constituted the strongest predictive factor for PDA in nAMD eyes in real-world clinical practice. The results of this study are a significant step toward image-guided prediction of long-term disease activity in the management of nAMD and highlight the importance of the automatic identification of photoreceptor layers.

## Introduction

Neovascular age-related macular degeneration (nAMD) is reported to be responsible for the majority of severe central vision loss in the elderly, and its prevalence is projected to rise as the population ages (Mitchell et al., 2018). Although the introduction of treatments targeting vascular endothelial growth factor (VEGF) for nAMD has offered remarkable anatomical and functional success, the best maintenance regimen of anti-VEGF therapy has been debated (Wykoff et al., 2015; Stoller et al., 2016; Dugel et al., 2020). In addition, nearly half of patients suffer from an incomplete response and repeated recurrence, despite receiving anti-VEGF therapy (Ying et al., 2014; Comparison of Age-related Macular Degeneration Treatments Trials (CATT) Research Group, Martin et al., 2020). In real-world clinical practice, early prediction of persistent disease activity (PDA) in an eye of an individual with nAMD, which was defined as persistent retinal fluid exudation, unresolved hemorrhage, and progressive fibrosis in spite of standardized anti-VEGF therapy (Mettu et al., 2021), is crucial for guiding treatment decisions and represents significant unmet needs in clinical.

Although spectral domain optical coherence tomography (SD-OCT) has become the first choice for assessing the lesion activity and the need for therapy of nAMD, manual segmentation and identification of pathognomonic and prognostic patterns are impractical in both clinical and research settings due to the high rates of discrepancies even among professional graders at the reading center, aside from the enormous amount of time and effort put in DeCroos et al. (2012), Toth et al. (2015). The application of deep learning (DL)-based algorithms has remarkably improved the efficiency and accuracy of pathological feature segmentation and provided more granular disease progression evaluations. Recently, DL systems have been introduced to detailed analyses on the morphologic features of SD-OCT scans of patients with nAMD and have found that the key imaging biomarkers associated with anti-VEGF treatment needs could be the presence of several pathologic changes, including intraretinal fluid (IRF), subretinal fluid (SRF), subretinal hyperreflective material (SHRM), and pigment epithelium detachment (PED) (Bogunovic et al., 2017; Holz et al., 2021).

In a number of studies, the role of the outer retina in the genesis and progression of nAMD has been documented (Dolz-Marco et al., 2017; Araujo et al., 2018). Due to the exceptional capacity to discern individual retinal layers, SD-OCT allows for in-depth study of the microstructural alterations in different layers of the retina in nAMD, notably the outer layers. The external limiting membrane (ELM), which is formed by continuous heterotypic junctions between Müller cells and photoreceptors, has been reported to contribute to fluid accumulation in the macula (Chen et al., 2012), while the ellipsoid zone (EZ), which on SD-OCT represents the inner segment/outer segment junction, is frequently utilized to evaluate photoreceptor pathology in a number of retinal diseases (Cuenca et al., 2020). Increasing studies have found that the integrity of ELM and EZ identified manually was associated with fluid response and visual function in patients with nAMD treated with either photodynamic therapy or anti-VEGF (Coscas et al., 2015; Woronkowicz et al., 2020). So far, the difficulty in reliably quantifying the often subtle contours is the fundamental drawback of the outer retinal bands as imaging indicators in nAMD. Hence, to increase our understanding of nAMD progression and treatment response, we need to be able to reliably and accurately characterize the condition of the ELM and EZ with great repeatability. However, there have been few studies that automatically identify and quantify SD-OCT imaging biomarkers, such as EZ and ELM, in order to investigate their relationship with PDA. Further work is needed to apply DL to predict long-term disease activity in eyes of patients with nAMD and achieve clinically useful prediction accuracy.

In this study, we presented a DL-based algorithm to simultaneously identify and quantify IRF, SRF, PED, SHRM, EZ, and ELM on SD-OCT scans, attempting to display the predictive value of anatomical parts, especially the integrity of ELM and EZ on the SD-OCT scan for PDA, and establishing a prediction model to recognize PDA from the eyes of individuals with nAMD during the initiation phase of the therapeutic course.

## Materials and methods

### Study participants

A total of 332 eyes of 261 patients with nAMD were treated with intravitreal injection of anti-VEGF drugs at the Qilu Hospital of Shandong University (QLHSDU) from 10 March 2016 to 26 May 2021 and were retrospectively analyzed. The criteria were ≥55 years old; patients with visual impairment due to active and untreated macular neovascularization (MNV) and were diagnosed according to the results of SD-OCT, fluorescein fundus angiography (FFA), or OCT angiography (OCTA), and, if necessary, indocyanine green angiography (ICGA); the patients had not received any treatment at the start of the 1-year interval; anti-VEGF drugs included aflibercept, ranibizumab, or conbercept; the therapeutic schedule was three consecutive monthly injections followed by *pro re nata* (3 + PRN), and treatment should be discontinued if the lesion disappears within 3 months. Patients with a history of other ocular disorders and previous operations were excluded. In the end, 186 eyes of 186 patients with nAMD were selected ultimately.

### Datasets and manual annotation

Our analysis included pre-therapeutic and post-therapeutic SD-OCT data obtained at baseline, month 1, month 3, and month 12. With RTVue XR Avanti devices (Optovue Inc., Fremont, CA, United States), SD-OCT B-scan images in an 18-line 8-mm radial macula pattern with the fovea as the scan center were collected, employing the in-built follow-up mode to enable pre- and post-therapeutic images to be scanned at the same location. To optimally benefit from the limited time and effort required for manual segmentation, one of the 18 radial lines from each B-scan was selected randomly to construct the dataset. Scans with unqualified image quality that cannot be properly segmented, such as strong noise, motion artifact, and shadow artifact, were excluded. The original resolution was 1,920 × 1,080 pixels. A total of 671 typical B-scans of 186 eyes were randomly selected and evaluated. The manual delineation process involved a total of 671 OCT scans, including 186 (27.7%) scans acquired at baseline, 186 (27.7%) scans at 1 month, 157 (23.4%) scans at 3 months, and 142 (21.2%) scans at 12 months. The manual annotation was provided by two expert ophthalmologists with 7 (Expert #1) and 25 (Expert #2) years of experience, from the Department of Geriatrics at Qilu Hospital of Shandong University, using Adobe Photoshop CS6 (Adobe Systems, San Jose, CA, United States). To ensure the high quality of annotation, we constructed the hierarchical labeling process: (1) Expert #1 was required to elaborately delineate the contours of target structures, and (2) Expert 2# should carefully review the annotation and revise the incorrect annotation. Before annotation, all graders were given a presentation on the relevant partitioning criteria. For objective comparison, we further divided the SD-OCT data into training and test sets at the patient level, with 148 patients for training and 38 patients for testing. The data for each patient in the test set included images taken from four time periods and ensured no overlap with the training set. During the training phase, the images from different patients and periods were randomly selected as the input of CNN and fully convolutional network (FCN) networks to enable the model to learn the characteristics of different periods. In other words, the networks were trained with 519 images, including 148 (28.5%) at baseline, 148 (28.5%) at 1 month, 119 (22.9%) at 3 months, and 104 (20.0%) at 12 months. The sensitive information of patients is de-identified. Review and analysis of retrospective anonymized data was approved by the Qilu Hospital Institutional Review Board and the research followed the principles of the Declaration of Helsinki.

### Algorithm architectures of automatic identification and segmentation

**FIGURE 1:**
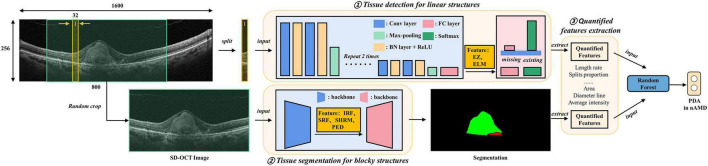
Algorithm architectures and training protocol. Deep learning (DL)-based methods were introduced to extract quantified features from original spectral domain optical coherence tomography (SD-OCT) as biomarkers. The algorithm includes three parts, namely, tissue detection for linear structures, including ellipsoid zone (EZ) and external limiting membranes (ELM); tissue segmentation for blocky structures including intraretinal fluid (IRF), subretinal fluid (SRF), subretinal hyperreflective material (SHRM), and pigment epithelium detachment (PED); final quantified features (e.g., the areas and diameter lines of IRF, SRF, SHRM, and PED) extraction from the above identification.

We introduced DL-based methods to extract a total of the six features associated with nAMD from original SD-OCT as biomarkers. The algorithm included three parts, namely, tissue detection for linear structures such as EZ and ELM; tissue segmentation for blocky structures such as IRF, SRF, SHRM, and PED; and final quantified features extraction from the above predictions (Figure 1).

The detection model of EZ and ELM was implemented using a classification convolutional neural network (CNN) model. The intuitive and effective solution was to split the SD-OCT images into slices and detected whether corresponding tissues exist in each slice. The training and test process of CNN followed clipping-training and clipping-predicting-merging-testing, respectively. Our CNN model was composed of four downstream modules, and each one was stacked with convolutional layers, batch normalization layers, and ReLU activation layers, followed by a max-pooling layer. The downstream modules extracted abstract features from medical images and down-sampled images to tensors. Then, two fully connected layers were adopted to reduce feature dimensions and then predicted the existence of the target. For the split whose prediction was positive, we defined that the EZ and ELM tissue was detected in the split. In specific, we employed the training dataset to train the model for the prediction of EZ and ELM, where each scan would be sliced into several 256 × 16 clips vertically. Then, the binary cross-entropy loss function was adopted to train CNN classifying the ELM or EZ on vertical clips. The final predictions of EZ and ELM can be merged from the corresponding vertical clips for testing.

For blocky structure tissues, we introduced a FCN to generate corresponding segmentation results, which meant predicting which tissue category each pixel belongs to. The FCN was composed of a backbone and a segmentation head. The performance of the backbone greatly affected the accuracy of segmentation results, and the Efficient Net was selected due to its balance of accuracy and efficiency. Due to the limitation of training samples, the backbone network was pretrained on ImageNet to leverage powerful prior knowledge for image understanding to accelerate the training process. The FCN took the data scan as input and manual delineation as the label for training. The final prediction was generated by the well-trained FCN model *via* feeding the test scan.

To help our detection and segmentation network understand the SD-OCT images better, we preprocessed samples uniformly. To be specific, we cropped the region of interest (ROI) of original SD-OCT images, reshaped them into a height of 256 and width of 1,600, and regularized the images to the range of min-max normalization. To mitigate the overfitting problem caused by limited training samples, some data augmentation methods were employed, such as random flip, random crop, and intensity shift. Based on the results of detection and segmentation, the biomarkers were extracted for linear structures EZ and ELM, and we focused on the length rate and the proportion of splits that target issue existed among all splits, while for blocky structures, we paid more attention to the shape, location, and intensity of tissues. For example, we calculated the area and diameter line of 4 tissues and, additionally, calculated the average intensity of SHRM and PED.

### Prediction models by random forest

**FIGURE 2:**
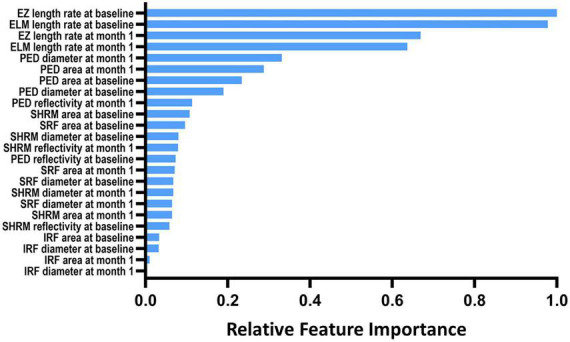
The relative importance of optical coherence tomography imaging biomarkers for the prediction of 1-year disease activity. IRF, intraretinal fluid; SRF, subretinal fluid; PED, pigment epithelial detachment; SHRM, subretinal hyperreflective material; EZ, ellipsoid zone; ELM, external limiting membranes.

Although the deep model achieves excellent performance in many downstream tasks, the amount of data on our nAMD prediction task is far less to meet the deep model requirements. In addition, logistics regression has a lower capacity and modeling ability that further limits the application in our future works on automatic nAMD typing based on multimodal images. On the contrary, the random forest model has high generalization capability and noise robustness. Therefore, in this study, the random-forest-based approach was employed to construct prediction models of the 1-year disease activity on nAMD eyes, based on the results of automatic segmentation of the extracted SD-OCT feature vectors listed in Figure 2. Each tree in the random forest makes an independent prediction, and we used majority voting to aggregate the predictions. We employed 10-fold cross-validation in our experiment. Then, we carried out the grid search experiment to select optimal hyper-parameters. The optimal prediction trees ranged from 150 to 800. To be specific, we evaluated three models, namely, two-metric (using EZ and ELM only), four-metric (IRF, SRF, SHRM, and PED), and six-metric (all six features). The differences between the corresponding features of multiple time points (baseline, baseline to 1 month, and baseline to 3 months) were also included to measure the rate of change of longitudinal features. 1-year disease activity was independently confirmed by two retinal specialists according to the results of SD-OCT and OCTA (FFA is not routinely performed after the initial treatments): Type 0 represents stable disease activity: SD-OCT shows the complete disappearance of retinal fluid; Type 1 represents PDA: persistent fluid exudation, or unresolved hemorrhage, or progressive fibrosis (Mettu et al., 2021); Type 2 represents cured: pathological lesions in the macular area (SHRM and PED) disappear without any type of retinal fluid. If the interpretation results of the two experts differed, they were instructed to reach an agreement through the guidance provided by the corresponding author (YQ), a more experienced retinal specialist, to reach an agreement.

## Statistical analyses

Basic statistical analyses were performed using SPSS statistical software (version 19.0; SPSS Inc.). The dice coefficient, accuracy, sensitivity, and specificity metrics were calculated for the automatic identification and quantification of lesions and microstructure in SD-OCT using Stats Models, version 0.6.1 (Python). The overlap rate between the manually labeled area and the automatically segmented area was evaluated by using the dice coefficient. Meanwhile, 95% confidence intervals (CIs) were provided. The prediction accuracy of all models was evaluated using a 10-fold cross-validation strategy and presented with the area under the receiver operating characteristic (ROC) curve (AUC), accuracy, sensitivity, and specificity.

## Results

### Data disposition and patient characteristics

A total of 671 typical B-scans were collected from 186 eyes of 186 patients with nAMD. The mean age was 75 years (SD 11.21), and 75.66% of the participants were men. After excluding useless cases, 671 typical B-scans were obtained and evaluated. Among them, 186 (27.7%) of the SD-OCT images were acquired at baseline, 186 (27.7%) at 1 month, 157 (23.4%) at 3 months, and 142 (21.2%) at 12 months, postoperatively. 1-year prognostic outcomes were obtained in 142 of 186 patients, including 66 eyes of type 0 (stable, 46.48%), 47 eyes of type 1 (recurrent or refractory, 33.10%), and 29 eyes of type 2 (cured, 20.42%). More clinical characteristics of patients are presented in Table 1.

**TABLE 1:** Baseline clinical characteristics.

**Age, years (SD)**	**75 (11.21)**
Male (%)	112 (75.66)
Right eye (%)	98 (52.69)
Baseline visual acuity (ETDRS letters, SD)	58.50 (14.32)
**Initial injectable drug (%)**	
Ranibizumab	82 (44.09)
Conbercept	91 (48.92)
Aflibercept	13 (6.99)
**One-year disease prognosis (%)**	
Type 0 (stable)	66 (46.48)
Type 1 (PDA)	47 (33.10)
Type 2 (cured)	29 (20.42)

SD, standard deviation; ETDRS, early treatment diabetic retinopathy study.

### Performance of automatic identification and segmentation

**FIGURE 3:**
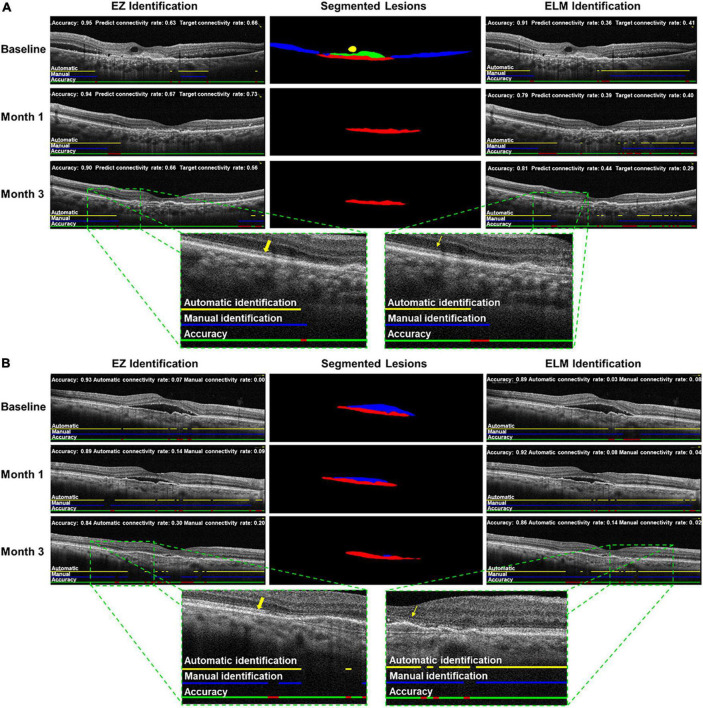
Examples of automatic identification and segmentation in neovascular age-related macular degeneration (nAMD) spectral domain-optical coherence tomography scan. Cases **(A,B)** at baseline, month 1, and month 3, including intraretinal fluid (IRF), subretinal fluid (SRF), pigment epithelium detachment (PED),subretinal hyperreflective material (SHRM), ellipsoid zone (EZ), and external limiting membranes (ELM) were automatically identified and segmented by trained deep learning (DL) algorithms. Left: automatic identification of EZ (yellow arrow); middle: automatic segmentations of IRF (yellow), SRF (blue), SHRM (green) and PED (red); right: automatic identification of ELM (yellow straight arrow).

Table 2 shows the performance metrics of the automatic identification and segmentation method. The dice coefficients of four lesions including IRF, SRF, SHRM, and PED ranged from 0.810 (95% CI: 0.778–0.842) to 0.949 (95% CI: 0.925–0.973), and the mean dice coefficient was 0.873 (95% CI: 0.847–0.899). The accuracy of all lesions, including those in the photoreceptor layer, ranged from 0.857 (95% CI: 0.841–0.873 for PED) to 0.989 (95% CI: 0.983–0.994 for IRF). The imaging biomarkers’ mean accuracy was 0.930 (95% CI: 0.916–0.943), with SRF at 0.976 (95% CI: 0.973–0.978), SHRM at 0.951 (95% CI: 0.940–0.962), EZ at 0.930 (95% CI: 0.908–0.952), and ELM at 0.873 (95% CI: 0.849–0.898), respectively. The sensitivity for all imaging biomarkers ranged from 0.798 (95% CI: 0.760–0.884 for SHRM) to 0.934 (95% CI: 0.906–0.962 for EZ), with comparable values for specificity (Table 2). Figure 3 shows an example of segmentation.

**TABLE 2:** The performance metrics of the automatic identification and segmentation methods.

	**IRF**	**SRF**	**SHRM**	**PED**	**EZ**	**ELM**	**AVG**
Accuracy (95% CI)	0.989 (0.983−0.994)	0.976 (0.973−0.978)	0.951 (0.940−0.962)	0.857 (0.841−0.873)	0.930 (0.908−0.952)	0.873 (0.849−0.898)	0.930 (0.916−0.943)
Sensitivity (95% CI)	0.813 (0.763−0.863)	0.887 (0.864−0.910)	0.798 (0.760−0.884)	0.886 (0.872−0.901)	0.934 (0.906−0.962)	0.918 (0.898−0.938)	0.873 (0.844−0.910)
Specificity (95% CI)	0.998 (0.997−0.998)	0.998 (0.997−0.998)	0.996 (0.995−0.998)	0.996 (0.995−0.997)	0.858 (0.805−0.911)	0.688 (0.640−0.737)	0.922 (0.905−0.940)
Dice coefficients (95% CI)	0.949 (0.925−0.973)	0.892 (0.873−0.911)	0.810 (0.778−0.842)	0.840 (0.811−0.870)	N/A	N/A	0.873 (0.847−0.899)

IRF, intraretinal fluid; SRF, subretinal fluid; PED, pigment epithelial detachment; SHRM, subretinal hyperreflective material; EZ, ellipsoid zone; ELM, external limiting membranes; AVG, average; 95% CI, 95% confidence interval.

### Relevance of spectral domain optical coherence tomography-derived features

Figure 2 depicts the top 24 essential imaging features ranked by absolute weight in the predictive model discovered. The features extracted at the baseline and 1-month visit were more relevant than those measured at 3 months 1. The integrity of EZ and ELM at both baseline and 1 month exhibited the strongest correlation with 1-year disease activity in nAMD of all features studied, according to the resulting ranking of feature relevance. The area, maximum diameter, and reflectivity of PED also played a moderate role in prediction, whereas parameters quantifying IRF exhibited less to no link with 1-year disease activity in a meaningful way.

### Quantitative analysis and prediction model

**FIGURE 4:**
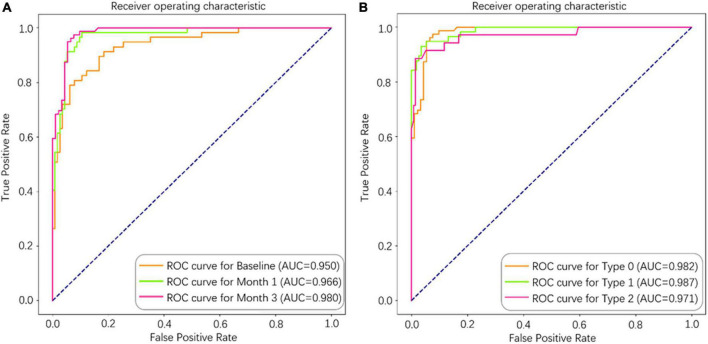
The area under the curve (AUC) of the deep learning (DL)-based model for predicting 1-year disease activity in neovascular age-related macular degeneration (nAMD). **(A)** Separate models were constructed from different longitudinal series of spectral domain optical coherence tomography (SD-OCT) data (baseline, baseline to month 1, and baseline to month 3). **(B)** Type 0 (stable), Type 1 (PDA), and Type 2 (cured) were predicted accurately in the 3-month model.

As shown in Table 3, the performance metrics of prediction models for 1-year disease activity grew monotonically with each month’s postoperative data put into the dataset. The baseline model achieved an AUC of 0.948, an accuracy of 0.871, a sensitivity of 0.834, and a specificity of 0.932. In contrast, the 3-month model (the model containing data from baseline to month 3) demonstrated the optimal predictive power with an AUC of 0.980, an accuracy of 0.930, a sensitivity of 0.920, and a specificity of 0.962 (Table 3 and Figure 4). The three categories of prognosis, type 0 (stable), type 1 (PDA), and type 2 (cured), were predicted accurately in the 3-month model with AUCs of 0.982, 0.987, and 0.971, respectively (Figure 4). The performance metrics of baseline, 1- and 3-month prediction models were significantly decreased when EZ and ELM information were excluded (Table 3). For example, the 3-month model including the EZ and ELM integrity data vs. excluding the data achieved an AUC of 0.980 vs. 0.950, an accuracy of 0.930 vs. 0.860, a sensitivity of 0.920 vs. 0.847, and a specificity of 0.962 vs. 0.925, respectively (Table 3).

**TABLE 3:** The performance metrics of separate prediction models based on the different combinations of biomarker variables (IRF, SRF, SHRM, PED, EZ, and ELM) and longitudinal time points (baseline, baseline to month 1, and baseline to month 3).

	Baseline			Month 1			Month 3		
	Six-metric model (using all variables)	Four-metric model (IRF, SRF, SHRM, and PED)	Two-metric model (EZ and ELM only)	Six-metric model (using all variables)	Four-metric model (IRF, SRF, SHRM, and PED)	Two-metric model (EZ and ELM only)	Six-metric model (using all variables)	Four-metric model (IRF, SRF, SHRM, and PED)	Two-metric model (EZ and ELM only)
AUC	0.950	0.902	0.826	0.966	0.912	0.872	0.980	0.950	0.875
Accuracy	0.871	0.795	0.713	0.912	0.795	0,772	0.930	0.860	0.778
Sensitivity	0.833	0.760	0.633	0.885	0.774	0.693	0.920	0.847	0.708
Specificity	0.932	0.889	0.856	0.953	0.892	0.887	0.962	0.925	0.887

AUC, area under the receiver operating characteristic curve.

## Discussion

In this study, we demonstrated a DL-based approach to identifying imaging biomarkers and developed a random forest model to predict PDA in the eyes of individuals with nAMD using a longitudinal series of SD-OCT scans obtained during the initiation phase. At the first stage, we used the classification CNN and segmentation FCN models to automatically recognize and quantify the SD-OCT features related to nAMD, such as the integrity of EZ and ELM, the area and diameter of IRF and SRF, as well as the area, diameter, and reflectance of PED and SHRM, and obtained good results with high accuracy. For the segmentation of SD-OCT images, we exploited the deep neural network to extract quantified features, overcoming the hurdles associated with heterogeneous reflectivity or variable morphologies with noise by packing them into a small area. The EZ and ELM layers were recognized simultaneously, avoiding the identification errors that are prevalent around these defect zones and difficult to interpret even for experienced readers (Loo et al., 2020). In addition, the technique for locating the inferior border of PED was modified to avoid segmentation errors when the basement membrane was unclear (Xu et al., 2017).

Then, based on the extracted quantitative features, the correlation between various SD-OCT image characteristics in different periods and PDA was investigated. Notably, in terms of predictions, the integrity of EZ and ELM constituted the strongest predictive factor in real-world clinical practice. Most research examined the correlation between visual acuity and the disruption of these two layers, but not how they correlated with the disease activity after anti-VEGF therapy in nAMD. As visual acuity also depends on interindividual differences, psychophysical properties, and the distance of the lesion from the macula, the anatomical response in clinical trials evaluating the effectiveness of anti-VEGF in patients with nAMD may be more convincing than visual acuity improvement. The current investigation emphasizes the distinctive relationship between outer retina damage and PDA in the eyes of patients with nAMD.

Despite the fact that the exact causes remain unknown, photoreceptors have been implicated to be the primary cellular effectors in the onset and progression of AMD. The epidemiological and genetic study has recently revealed that photoreceptor thinning, rather than retinal pigment epithelium (RPE) and Bruch’s membrane (BM) thickening, could be an early-stage biomarker for the likelihood of future AMD development (Zekavat et al., 2022). For nAMD, photoreceptor degeneration appears to be a secondary event to the loss of choroidal vasculature instead of presumably dysfunction in RPE (Kurihara et al., 2016). In addition, the current model of AMD pathogenesis in mouse has shown that metabolic stress of photoreceptors leads to the development of a complex pathology that affects overall RPE health over time (Cheng et al., 2020). The vicious cycle of choroidal blood flow decline, photoreceptor degradation, and RPE hypoxia seems to have the potential to accelerate the progression of nAMD. The integrity of ELM and EZ on SD-OCT also reflects the level of VEGF in previous studies of macular edema secondary to diabetics (Saxena et al., 2017), central retinal vein occlusion (Wolf-Schnurrbusch et al., 2011), and myopic choroidal neovascularization (Ding et al., 2018). Furthermore, the ELM was established as a barrier against macromolecules and dysregulated fluid dynamics, permitting the accumulation of IRF or SRF (Jain et al., 2013). The integrity of ELM may also reflect the precise architecture and organization of Müller cells, which provide a number of crucial support roles for appropriate photoreceptors and neuronal functions and contribute to inner retinal thickening (Derouiche et al., 2012). According to our findings, the disruption degree of ELM and EZ by the MNV lesion’s invasion and proliferation, which reflects the alterations of photoreceptors and RPE, could be a useful predictor for the PDA of nAMD at the treatment-naïve stage. Our results also demonstrated that the integrity of EZ and ELM significantly improved the performance of the six-metric model than that of the four-metric model, highlighting the importance of automatic identification of EZ and ELM in the prediction of PDA.

Surprisingly, IRF and SRF did not yield valid information regarding predicting long-term response to treatment, which was inconsistent with the study focused on early outcomes in the first 3 months after anti-VEGF therapy with an AUC of 0.666 (95% CI: 0.60–0.74) (Tan et al., 2020). One contributing factor to this gap may be the different time points for assessing PDA and the fact that the SD-OCT biomarkers in the above study only involved retinal fluid. Although the presence of fluid in the retina is a crucial factor in progressive functional loss in the real-world, new investigations have found that not all fluid phenomena seen with SD-OCT are connected to neovascular disease activity (Hilely et al., 2021; Jaffe et al., 2021). Specifically, the prediction model involved six SD-OCT imaging biomarkers that revealed a better performance than that of four biomarkers (IRF, SRF, PED, and SHRM) or two biomarkers (EZ and ELM), which achieved AUCs of 0.8 or 0.9; however, relatively lower accuracy and sensitivity. From a biological and imaging perspective, we speculate that the co-existence of these six SD-OCT imaging biomarkers emerges as the factor associated with the classification and PDA of nAMD, explaining the satisfactory performance of our prediction model. This association with clinical practice distinguishes our study from algorithm-focused studies, which are uninterpretable due to the “black box” nature of the DL model.

The most significant success is that this segmentation algorithm enabled us to analyze up to six indicators at once which is critical for building a predictive model. Based on the extracted quantitative features, our predictive model achieved optimal performance with an AUC of 0.980. This may assist ophthalmologists in adopting a treatment paradigm based on precision medicine, which contributes to patient counseling and injection decision-making. In addition, we achieve a promising prediction accuracy without the cost of interpretability and provide an effective prediction based on SD-OCT image information alone, which simplifies the clinical information acquisition process and may facilitate telemedicine and follow-up for nAMD with a primary healthcare system. Potential clinical uses of our DL-based method may include diagnosis and classification of nAMD.

Our DL-based algorithm shows a privileged performance on the prediction of PDA in the eyes of individuals with nAMD; however, several limitations must be considered. First, our study included a relatively small sample size with only Chinese patients; a modest follow-up period, a larger multiracial population, and a longer follow-up are needed for further validation. Second, our study only includes representative morphologic data from SD-OCT scans; additional clinical data, such as Best Corrected Visual Acuity (BCVA), age, diet, lifestyle, and genotyping, are not included. Furthermore, there was no external validation set, and the automatic recognition and segmentation of hyperreflective foci (HF), central subfield thickness (CST), and subfoveal choroidal thickness (SFCT), all of which have been shown to be prognostically significant in previous studies, were not included.

## Conclusion

This study automatically extracted imaging biomarkers on SD-OCT scans from the eyes of patients with nAMD by using classification CNN and segmentation FCN models. It also found that the disruption of ELM and EZ could be used as objective and reproducible imaging tools in the follow-up of patients with nAMD treated with anti-VEGF, and then constructed a prediction model on PDA from the eyes of individuals with nAMD by using the random forest algorithm. The model demonstrates high accuracy in long-term prognosis after anti-VEGF treatment. Further investigation is needed to improve the generalization capability of the model, contributing to a precise therapeutic strategy for the eyes of individuals with nAMD.

## Data availability statement

The original contributions presented in this study are included in the article/supplementary material, further inquiries can be directed to the corresponding author.

## Ethics statement

The study involving humans (retrospective anonymized data) was reviewed and approved by the Medical Ethics Committee of Qilu Hospital and the research followed the principles of the Declaration of Helsinki. Written informed consent was obtained from all participants for their participation in this study.

## Author contributions

XS and YQ were responsible for the concept and design. XS and QX collected and analyzed the data and wrote the original manuscript draft. MN, CB, and CY developed the algorithm. XS and MN were responsible for statistical analysis. XS, YZ, and ZZ revised the manuscript. YQ supervised the project. YQ and XS were the guarantors of this study, as such, had full access to all of the data in the study, take responsibility for the integrity of the data, and the accuracy of the data analysis. All authors contributed to data interpretation, critical revision of the manuscript, and approved the submitted version.
